# HARMFUL ALGAL BLOOMS: An Unexpected Deep-Sea Diver

**DOI:** 10.1289/ehp.117-a242a

**Published:** 2009-06

**Authors:** Carol Potera

Domoic acid (DA), a potent neurotoxin produced primarily by the diatom genus *Pseudo-nitzschia*, is generated during harmful algal blooms—rapid surges in toxic algae populations that result from increases in nutrient availability, temperature, and sunlight, among other environmental changes. Previously, scientists assumed that once the blooms dissipated, DA was released into and diluted within the upper ocean layer. But a study published in the April 2009 issue of *Nature Geoscience* shows that DA can be trapped inside the silica shells of *Pseudo-nitzschia* and carried to the ocean floor. Concentrations of DA in particles collected in deep oceans were several times higher than the regulatory limit set by the U.S. Environmental Protection Agency (EPA) to prevent human poisoning.

DA can cause a condition called amnesic shellfish poisoning in people who eat contaminated crabs, oysters, clams, mussels, scallops, anchovies, and sardines—all of which feed on *Pseudo-nitzschia*. Symptoms include gastrointestinal upset, headache, dizziness, cardiac arrhythmia, coma, potential loss of short−term memory, and possibly death. Water−soluble DA concentrates in the stomachs of shellfish and tiny fish. To ingest DA, people must eat the whole organism, including the stomach, says Stephen Bates, phytoplankton scientist emeritus with Fisheries and Oceans Canada.

Although elderly people are considered the most vulnerable to the effects of DA, early−life exposures also may be problematic. Recent rodent studies by biologists at the University of Prince Edward Island, published in the 23 March and 16 April 2009 issues of *Physiology & Behavior*, found that neonatal exposure to low doses of DA was associated with lasting cognitive deficits and behavioral problems in adult animals. Moreover, findings reported in the December 2008 issue of *Marine Drugs* suggest DA may be immunotoxic in mice.

For the current study, researchers set up sediment traps off the coast of Southern California, where *Pseudo-nitzschia* blooms and DA poisoning are prevalent. The traps floated above the sea floor at depths of 540, 550, and 800 m. Sediment collected in traps set at 550 m contained up to 50 μg DA/g dry sediment, and traps set at 800 m contained up to 163 μg DA/g of dry sediment. Measurements showed that DA sank rapidly, settling to 800 m in about three days. Bates says shellfish and sediment levels are not directly comparable because the former reflects wet weight, whereas the latter reflects dry weight, but that a rough comparison can be made for the purpose of assessing relative amounts.

The findings suggest that marine creatures living in deeper waters may be contaminated with DA, yet health officials currently monitor only shellfish that live close to the surface. The U.S. EPA and the Canadian Food Inspection Agency regularly check commercial shellfish beds, with increased testing during algal blooms, closing the beds when levels reach 20 μg DA/g tissue. However, says study leader Claudia Benitez−Nelson, a geochemist at the University of South Carolina, “We no longer can use algal blooms as an indicator of [potential] DA poisoning.”

The fact that DA sinks to deeper waters may, in fact, help explain past mysterious outbreaks of shellfish poisoning. For instance, in 1995 lucrative deep−sea scallop beds were closed to harvesting off the coast of Nova Scotia in the Gulf of Maine. The scallops contained up to 3,400 μg DA/g tissue. “We didn’t know where the DA came from,” Bates says. “But the new data suggest that the cause could have been DA sinking down from surface blooms of *Pseudo-nitzschia*.”

The key to curbing DA poisoning is to understand why and when *Pseudo-nitzschia* blooms occur. Although harmful algal blooms are mainly viewed as natural phenomena, the magnitude and occurrence of some toxic species can be exacerbated by nutrient inputs from human sewage and fertilizer and possibly other forms of coastal pollution. “People are working hard to reduce runoff from crops and lawns, but it takes time,” says Benitez−Nelson. Meanwhile, she adds, “Once [*Pseudo-nitzschia*] bloom, it’s very difficult to control the toxins they produce.”

